# Antiviral effect of copper chloride on feline calicivirus and synergy with ribavirin in vitro

**DOI:** 10.1186/s12917-020-02441-0

**Published:** 2020-07-06

**Authors:** Dengliang Li, Zhanding Cui, Guohua Li, Liangting Zhang, Ying Zhang, Han Zhao, Shuang Zhang, Yanbing Guo, Yanli Zhao, Fanxing Men, Shihui Zhao, Jiang Shao, Dongju Du, Hailong Huang, Kai Wang, Guixue Hu, Tiansong Li, Yongkun Zhao

**Affiliations:** 1 College of Animal Science and Technology, Jilin Agricultural University, Changchun, 130118 China; 2 Institute of Military Veterinary Medicine, Academy of Military Medical Sciences, 666 Liuying West Road, Changchun, 130122 China; 3 College of Animal Science and Technology, Shihezi University, Shihezi, 832003 China; 4 College of Continuing Education, Hebei Agricultural University, Baoding, 071001 China; 5 College of Wildlife and Protected Area Northeast Forestry University, Harbin, 150040 China; 6 Animal Husbandry and Veterinary Science Research Institute of Jilin Province, Changchun, 130062 China; 7 College of Science, Beihua University, Jilin, 132013 China

**Keywords:** Feline calicivirus, Copper chloride, Antiviral effect, Synergistic protective effect, Antagonistic effect

## Abstract

**Background:**

Feline calicivirus (FCV) is a common and highly prevalent pathogen causing upper respiratory diseases in kittens and felines in recent years. Due to the substantial genetic variability of the viral genes, existing vaccines cannot provide complete protection. Therefore, research on FCV antiviral drugs has received much attention.

**Results:**

In this study, we found that copper chloride had dose-dependent antiviral effects on FCV in F81 cells. We also found that the combination of copper chloride and ribavirin had a synergistic protective effect against FCV in F81 cells. In contrast, the combination of copper chloride and horse anti-FCV immunoglobulin F (ab’)_2_ showed an antagonistic effect, likely because copper chloride has an effect on F (ab’)_2_ immunoglobulin; however, further research is needed to clarify this supposition.

**Conclusions:**

In summary, we found that copper chloride had low cytotoxicity and significant antiviral effects on FCV in F81 cells, providing a new drug candidate for the prevention and treatment of FCV infection.

## Background

Feline calicivirus (FCV) is a small, nonenveloped, single positive-strand RNA virus that belongs to the genus *Vesivirus* of the family Caliciviridae [1]. Most felines (e.g., cats, tigers, and cheetahs) are susceptible, and infection in dogs has been reported in recent years [2, 3]. Kittens aged 1 to 12 weeks are mainly infected, which results in a mortality rate of 67% [4, 5]. Infected animals present with oral ulcers, chronic stomatitis, rhinitis, conjunctivitis, and pneumonia [6–9]. The primary prevention method at present is vaccination. However, due to the high evolutionary rate of the FCV capsid protein, which results in as many as 1.3 × 10^− 2^ to 2.6 × 10^− 2^ substitutions per nucleotide per year [10], traditional vaccination cannot completely protect animals from reinfection with mutant or wild-type strains, even though FCV is considered to have only one serotype [11]. Therefore, it is necessary to develop a safe and effective antiviral drug as a monotherapy or as part of a combination treatment.

In previous studies, various compounds had been found to have anti-FCV effects, such as minomorpho oligophosphate (PMO), lithium chloride, and germacrone [12–14]. The combination of mefloquine and recombinant cat interferon-ω had a synergetic effect against FCV [15]. Copper is an indispensable element used in the production of livestock and poultry. Basic copper chloride is an essential additive in feed [16]. Researchers previously found that copper and copper compounds had antiviral effects against dengue virus, influenza virus, and human immunodeficiency virus (HIV) in vitro [17–19]. A research report on the cytotoxicity of copper chloride showed that the cytotoxicity of copper chloride was minimal in the range of cytotoxic concentrations that did not disrupt the stability of copper [20]. The copper (II) chloride complex has also been utilized for its anticancer effects in vitro on human cervical cancer, colon cancer, ovarian cancer, and melanoma cell lines [21]. However, there have been no reports of the antiviral effect of copper chloride against FCV.

In this study, we found that copper chloride had low cytotoxicity in F81 cells. We evaluated the antiviral effects of copper chloride in vitro in a dose-dependent manner by measuring the TCID_50_ and using RT-qPCR to analyse the effects against FCV. Additionally, the combination of copper chloride and ribavirin showed synergistic antiviral effects against FCV.

## Results

### Cytotoxicity test of copper chloride in F81 cells

**Fig. 1:**
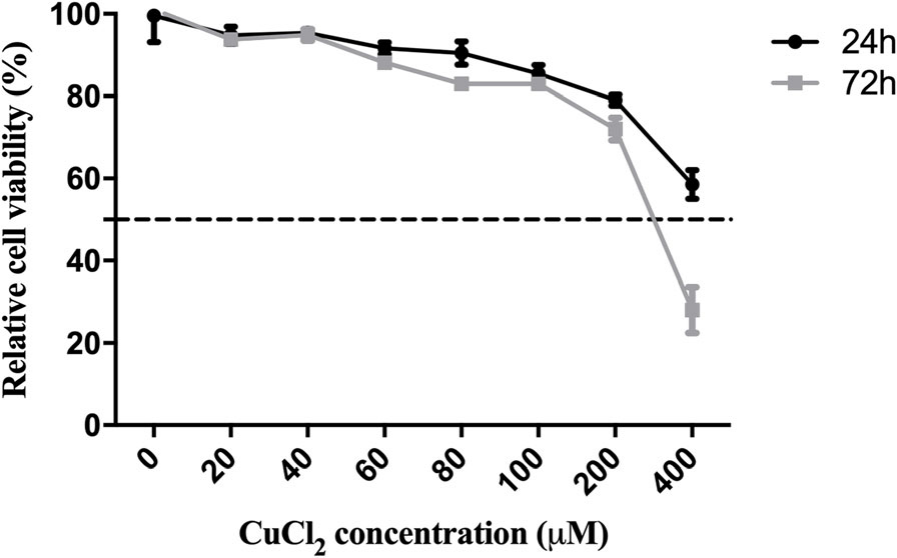
Cytotoxicity assay of copper chloride in F81 cells. A CCK-8 assay was used to measure cytotoxicity in F81 cells exposed to copper chloride at concentrations of 400, 200, 100, 80, 60, 40, and 20 μM during incubation at 37 °C in 5% CO_2_ for 24 h or 72 h. The relative activity of 0.4% DMSO-treated F81 cells was considered to be 100%, and the cytotoxicity is shown as the percentage of cell activity with respect to that of cells subjected to the DMSO mimetic treatment. Each value represents three independent replicate experiments

The results of the cytotoxicity assay showed that the relative cell viability was greater than 85% after treatment with copper chloride at a concentration of 20 ~ 80 μM for 24 h or 72 h. When the concentration of copper chloride was less than or equal to 200 μM (below the CC_50_), the relative cell viability of the F81 cells was higher than 50% after 24 h or 72 h of treatment, indicating that copper chloride can be regarded as nontoxic in this concentration range (Fig. 1). Therefore, a concentration of 200 μM copper chloride was used as the maximum concentration for the antiviral experiments.

### Antiviral effect of copper chloride at different doses

**Fig. 2:**
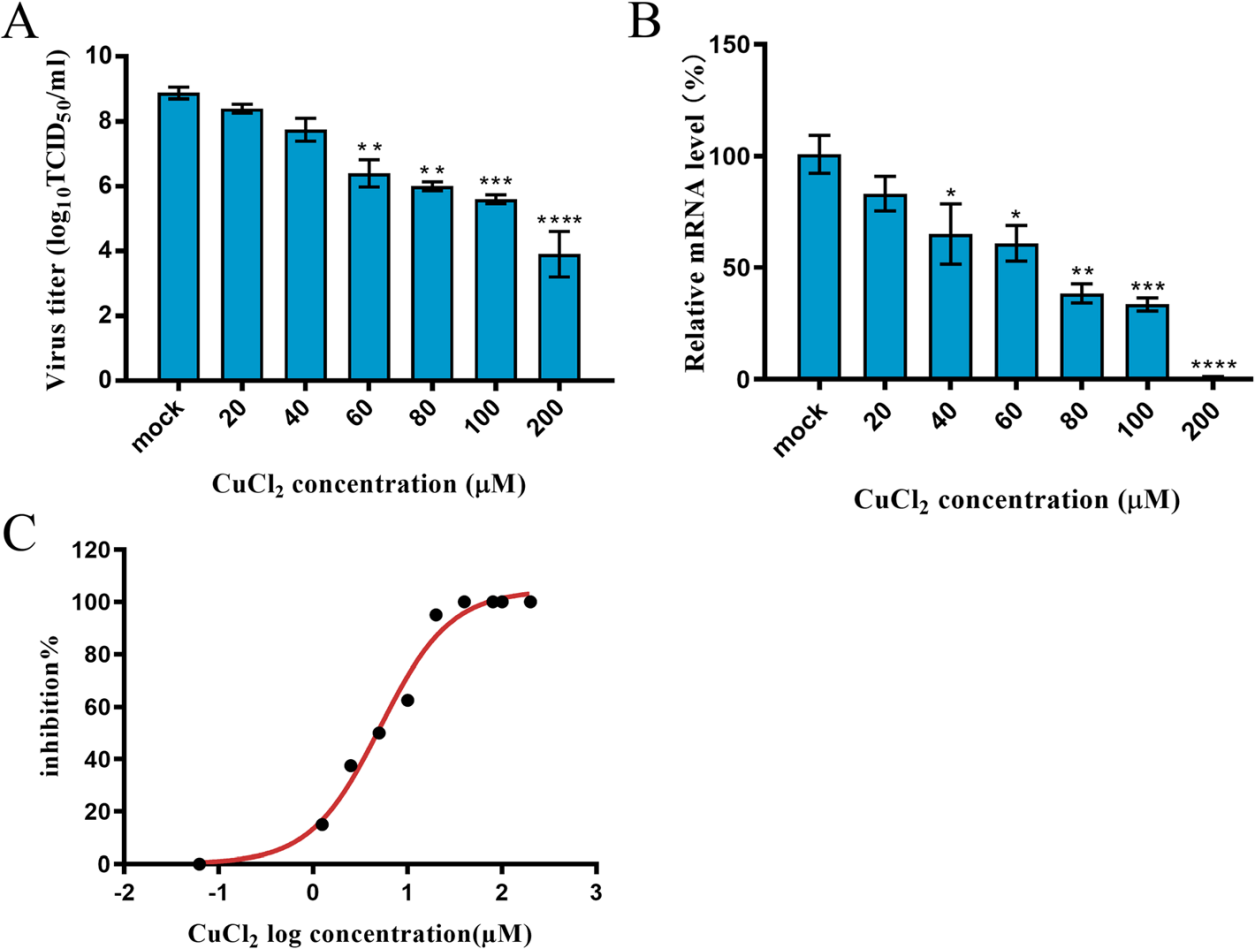
The antiviral effect of different doses of copper chloride (20–200 μM) on F81 cells infected with FCV at 100 TCID_50_ . After incubation for 28 h at 37 °C in 5% CO_2_, the virus titre (**a**) and the relative RNA levels (**b**) of FCV were detected. **c** The IC_50_ of copper chloride for FCV was determined; * *p* < 0.0332; ** *p* < 0.0021; *** *p* < 0.0002; and **** *p* < 0.0001

To evaluate the antiviral effects of different doses of copper chloride on FCV, we examined the virus titer and RNA levels of FCV in F81 cells treated with different doses of copper chloride. The results showed that the virus titre was significantly lower than that of the mock group in cells exposed to 60, 80, 100, and 200 μM concentrations of copper chloride (*p* < 0.01) (Fig. 2a). The relative RNA levels of FCV were significantly reduced compared to those of the mock group when the copper chloride concentration was 80 or 100 μM (*p* < 0.05), and the decrease was highly significant at 200 μM copper chloride (*p* < 0.001) (Fig. 2b). In addition, all the results indicated that the antiviral effect of copper chloride on F81 cells was dose-dependent. The IC_50_ of copper chloride for FCV was determined to be 5.1 μM (Fig. 2c).

### Indirect immunofluorescence assay

**Fig. 3:**
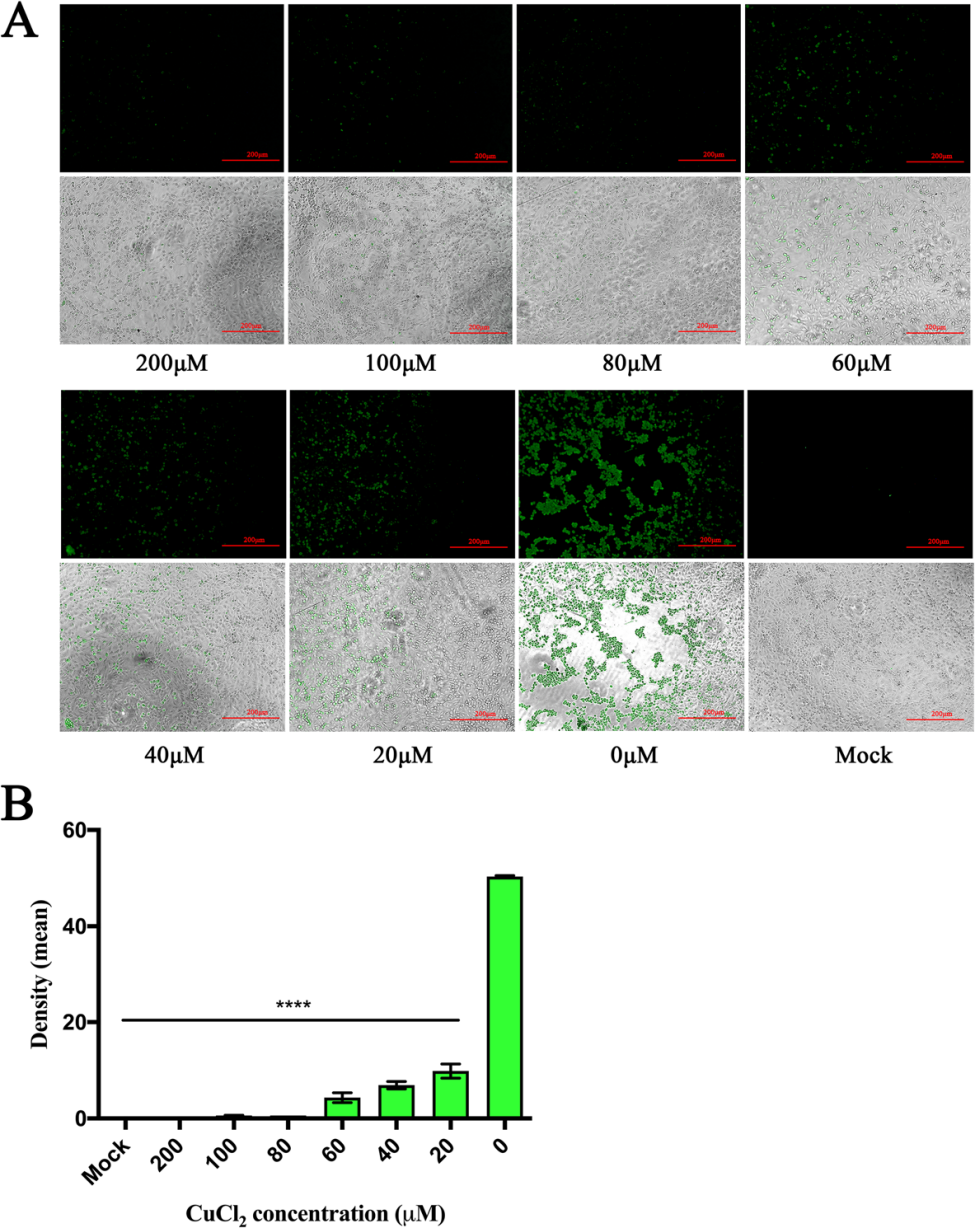
An IFA verified the antiviral effect of copper chloride. **a** F81 cells were infected with different concentrations of copper chloride (20–200 μM) and FCV (100 TCID_50_), and a mock treatment group containing 0.4% DMSO and a negative control group that was not infected with FCV were used as controls. After incubation for 1 h at 37 °C in 5% CO_2_, an IFA of the F81 cells was performed. **b** Two experimental wells were selected in the 96-well cell plate, and the fluorescence signals were detected in randomly selected areas in each well using ImageJ software. The experimental result was expressed as the average of two fluorescence signals. The antiviral effect of copper chloride was evaluated by detecting the fluorescence signal; **** *p* < 0.0001

To further evaluate whether the antiviral effect of copper chloride on FCV was dose-dependent, we performed an indirect immunofluorescence assay (IFA). The results showed that an intense green fluorescence signal was observed in the 0 μM group, and only weak fluorescence signals were observed at concentrations of 60, 40, and 20 μM, indicating a dose-dependent relationship after greyscale scanning (Fig. 3b). There was almost no fluorescence signal at concentrations of 80, 100, and 200 μM or for the control group compared to that of the mock group (Fig. 3a). In addition, the F81 cells treated with 200 μM copper chloride showed slight rounding and polymerization. This finding also validates the previously described range of the nontoxic concentrations of copper chloride.

### Antiviral effect of copper chloride for different treatment times

**Fig. 4:**
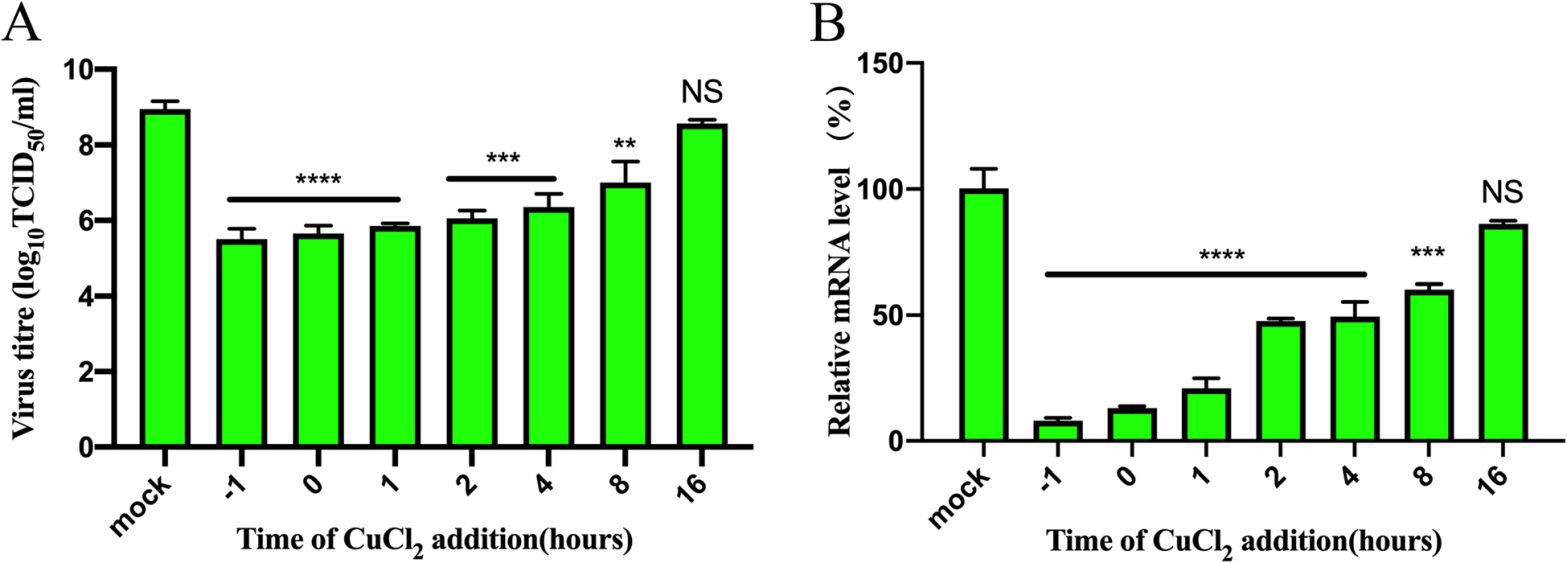
The antiviral effect of copper chloride on FCV at different time points. F81 cells were infected with 100 TCID_50_ FCV and then treated with 80 μM copper chloride for − 1, 0, 1, 2, 4, 8, and 16 h. After incubation for 24 h at 37 °C in 5% CO_2_, the virus titre (**a**) and relative RNA levels (**b**) of FCV were detected. NS, *p* > 0.1234; ** *p* < 0.0021; *** *p* < 0.0002; and **** *p* < 0.0001. (‘-1 h’ indicates that the cells were treated with CuCl_2_ 1 h before infection)

To evaluate the antiviral effect of copper chloride on FCV for different treatment times, we examined the virus titer and RNA levels of FCV in F81 cells for different treatment times. The experimental data showed that 80 μM copper chloride had good antiviral effects on FCV and decreased cytotoxicity. Therefore, we selected this concentration of copper chloride for subsequent experiments. The results showed that the virus titre and the relative RNA level of FCV were significantly lower in the copper chloride treatment group after − 1, 0, and 1 h of FCV infection than those in the mock group (*p* < 0.001), and there was also a significant difference after 4 and 8 h of infection (*p* < 0.002). After 16 h of virus infection, there were no significant differences between the virus titre or relative RNA level of the FCV and the mock group, indicating that the antiviral effect of copper chloride (80 μM) was not significant after 16 h of FCV infection (Fig. 4a and b). The RT-qPCR data also reflected similar results. Thus, the antiviral effect of copper chloride on FCV was mainly observed in the early stages of virus proliferation.

### Antiviral effect of copper chloride on different strains of FCV

**Fig. 5:**
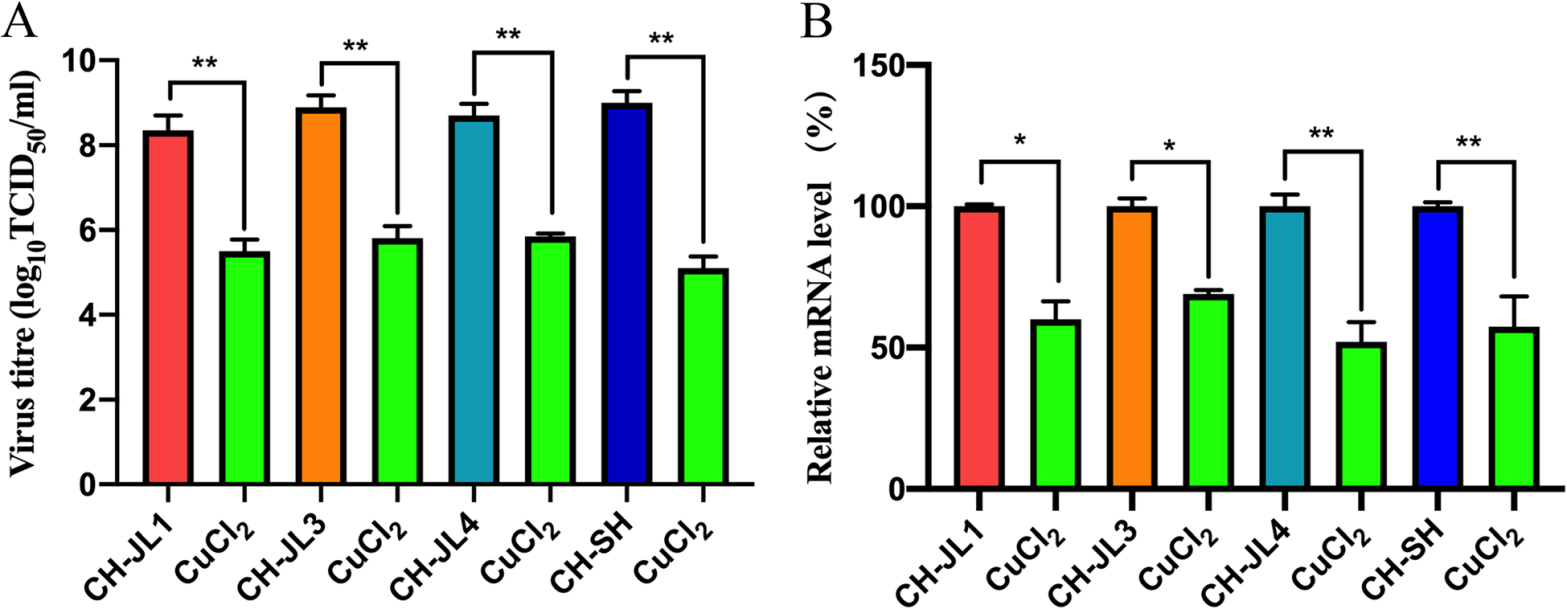
The antiviral effect of copper chloride on different FCV strains. The F81 cells were infected with different FCV strains at 100 TCID_50_ and treated with 80 μM copper chloride. After incubation for 28 h at 37 °C in 5% CO_2_, the virus titre (**a**) and the relative RNA levels (**b**) of FCV were detected; * *p* < 0.1234 and ** *p* < 0.0021

For other strains of FCV, the antiviral effects of copper chloride were validated. As previously described, we determined the viral titer and relative viral RNA levels of the different strains after copper chloride treatment. The results showed that the use of 80 μM copper chloride to treat other strains of FCV (CH-JL1, CH-JL3, CH-JL4, and CH-SH) significantly reduced the viral titre and relative viral RNA levels of the infected F81 cells (Fig. 5a and b). These results indicated that copper chloride had a strong antiviral effect against strains of FCV with differing genotypes.

### Synergistic effect of ribavirin and the antagonistic effect of F (ab’)_2_

**Fig. 6:**
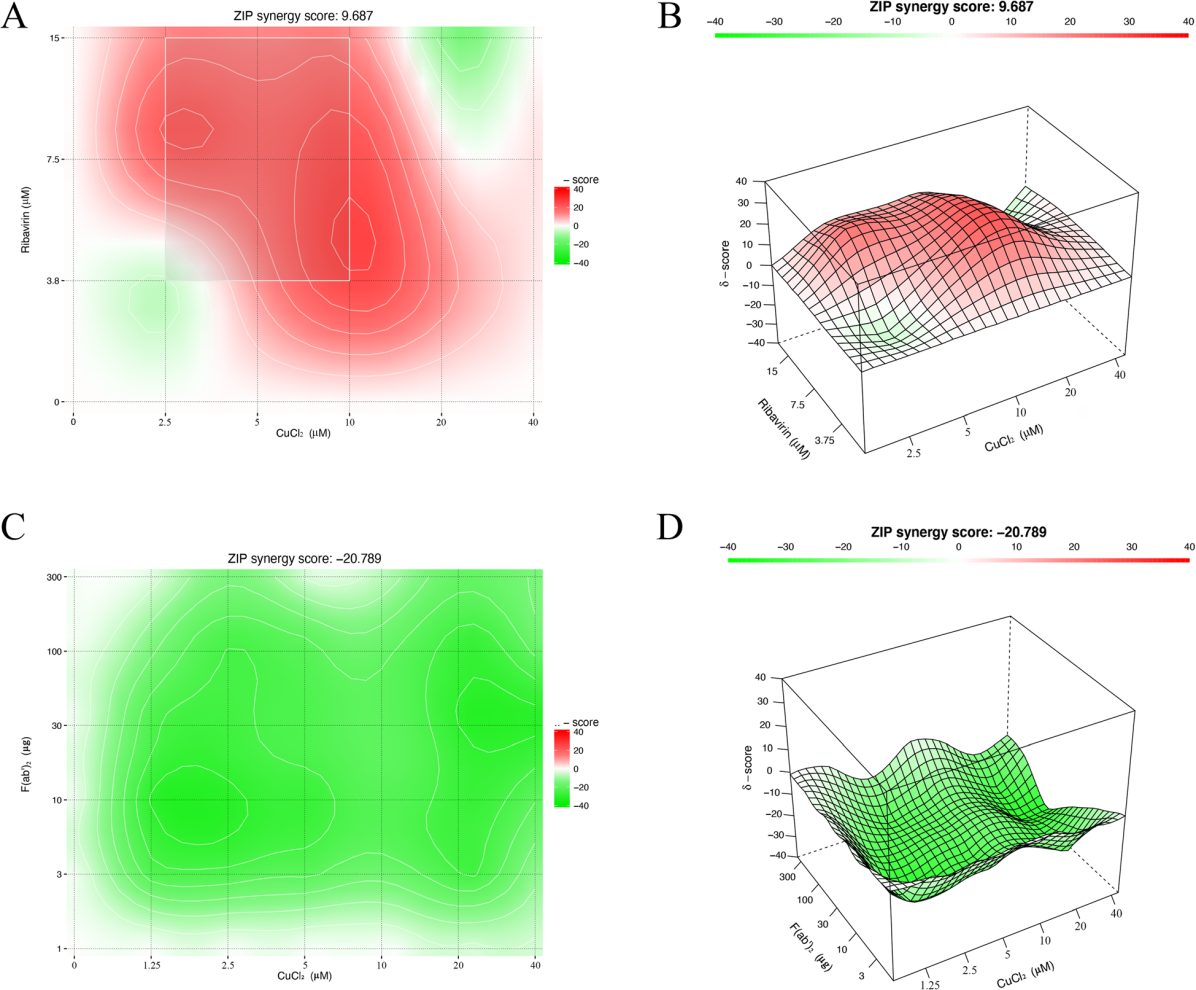
The antiviral effect of the compound combination. **a** and **c** The compounds were diluted to the indicated concentrations and used in combination to treat FCV infection. The results of the RT-qPCR were statistically analysed by the methods described above, and the effects of the drug combination were evaluated using SynergyFinder. **b** and **d** The interaction scores for different concentrations of compounds were calculated using the ZIP model. The synergy score for the ZIP model was expressed as the average of all δ scores for the dose-response landscape, and the red portion of the graph indicates synergy. All experiments were repeated three times

The checkerboard method was used to mix different compounds, and the antiviral effect of the compound combinations were evaluated using SynergyFinder. The results showed that the combination of copper chloride and ribavirin had a synergistic protective effect on FCV-infected F81 cells within a specific concentration range (Fig. 6a). The ZIP model was used to calculate the average score of the synergy of the two compounds, and it was found to be 9.687 (Fig. 6b). Conversely, the combined use of copper chloride and F (ab’)_2_ showed antagonistic effects, and the average score of the two compounds calculated by the ZIP model was − 20.798 (Fig. 6c and d).

## Discussion

In previous studies, copper or copper ions were shown to have an antiviral effect on herpes simplex virus (HSV) [22]. However, they were mainly used to inactivate virions since copper ions can disrupt the activity of certain proteins containing hydroxyl radicals [21, 23]. However, when the concentration of copper ions was too high, the resulting toxicity was fatal because copper chloride can inactivate specific proteins and produce a large number of reactive oxygen species in cells to induce apoptosis [24]. In our study, we found that low concentrations of copper chloride induced low levels of cytotoxicity in F81 cells. The F81 cells treated with copper chloride concentrations up to 200 μM for 24 h and 72 h showed cell activity greater than 50%, which was similar to that previously reported for the cytotoxicity of soluble copper chloride. Therefore, the cytotoxicity of copper chloride was very low within the range of cytotoxic concentrations that did not disrupt the stability of copper [20]. However, the safety of copper chloride administration to animals remained to be further explored. Previously, copper chloride dihydrate treatment exhibited excellent antiviral activity against dengue virus, but the antiviral effect of copper chloride on FCV had not been reported [25]. In this study, we found that copper chloride had an antiviral effect on FCV with an IC_50_ of 5.1 μM. The IFA results showed that the inhibition of FCV by copper chloride was dose-dependent and significantly reduced the cytopathic effect (CPE) of FCV on F81 cells. After treatment with different concentrations of copper chloride, the viral titer and RNA expression levels of FCV in cells were significantly different from those in the control cells.

Then, we demonstrated the inhibitory effect of copper chloride on the viral titre and RNA levels of FCV at different time points. FCV replication was significantly inhibited when copper chloride was added 8 h after FCV was used to infect cells, but copper chloride did not significantly inhibit FCV after 16 h. Our findings were similar to those of previous studies using fexaramine, LiCl, ginger extracts, or CSX [13, 14, 26, 27]. We speculated that copper chloride could hinder the attachment and entry of FCV and inhibit replication inside the cell, but the mechanism involved in inhibiting replication needs further verification. Past research has shown that copper ions have the ability to disrupt DNA through cross-linking with single-stranded polynucleotide chains as well as forming intra- and intra-strand cross-links [26]. Numerous studies have reported that copper ions can inhibit neuraminidase (NA) in cells and tissues [27, 28]. Both influenza virus NA [17, 29] and Sendai virus NA [30] could be inhibited by copper, but it had not been determined whether the antiviral effect was related to NA inhibition. The copper ions resulted in the abnormal morphology of the virus, which might indicate that the structural changes could be related to antiviral effects [17]. We found that copper chloride also inhibited the replication of other FCV strains in F81 cells, which was further evidence of the antiviral activity of copper chloride against FCV. The ZIP model in SynergyFinder was used to analyse the combined effects of the drugs. The synergy score for the ZIP model was expressed as the average of all the δ scores in the dose-response landscape [25, 31]. In a later experiment, we found that the broad-spectrum antiviral drug ribavirin and copper chloride had synergistic effects on inhibiting FCV. The most synergistic area had a score of 15.5, with an average score of 9.7. However, copper chloride and immunoglobulin F (ab’)_2_ showed a robust antagonistic effect. It has been reported in the literature that copper ions could induce IgG cleavage [32, 33]. Past research has found that when metal ions with a specific stereochemistry in the appropriate conformation are present, metal ions can increase the rate of peptide cleavage and catalyse the reaction [34, 35]. In fact, copper ions could bind and enhance the degradation of proteins such as monoclonal antibodies and hydrolyse small peptides and bovine serum albumin, and trace amounts of copper ions might be sufficient to enable site-specific, metal-mediated monoclonal antibody degradation [36]. When copper chloride and immunoglobulin F (ab’)_2_ are applied to F81 cells infected with FCV at the same time, there might be mutual effects that cause antagonistic activity. However, further research is required to prove this hypothesis.

## Conclusions

In summary, we verified that copper chloride had low cytotoxicity in F81 cells and had an antiviral effect on FCV in vitro. Later, we found that copper chloride had an effect on the replication stage in FCV-infected cells, and it had a synergistic effect with ribavirin, but drug resistance should be determined in a future study. This study indicated that copper chloride could be a promising candidate drug for the treatment of FCV infection in felines.

## Methods

### Viruses, cells and compounds

The FCV CH-SH strains and F81 cells were provided by the Institute of Military Veterinary Medicine, Academy of Military Medical Science. FCV CH-JL1/CH-JL2/CH-JL3/CH-JL4 were isolated and stored in our laboratory [37]. The horse anti-FCV immunoglobulin F (ab’)_2_ was produced and stored in our laboratory [38]. The copper chloride dihydrate (Aladdin, China) product number was C111685. The ribavirin (Aladdin, China) product number was R101754.

### Cytotoxicity assay for copper chloride

Copper chloride was diluted in DMSO to a concentration of 100 mM as a stock solution, and the stock solution was diluted to different concentrations (400 μM, 200 μM, 100 μM, 80 μM, 60 μM, 40 μM, and 20 μM) with MEM containing 2% FBA. The cells were incubated at 37 °C in 5% CO_2_ for 24 h or 72 h. After washing the cells twice with PBS solution, 180 μL of FBA-free MEM and 20 μl of CCK-8 reagent (CCK-8; Dojindo, Japan) were added to each well. After incubation at 37 °C for 1–2 h, the optical density (OD) was determined at 450 nm using a Cmax Plus microplate reader (Molecular Devices, USA). The cell viability was calculated by the formula [OD_450_ (compound) - OD_450_ (blank)]/[OD_450_ (control) - OD_450_ (blank)] × 100%. The copper chloride concentrations that were less than 50% of the cytostatic concentration (CC_50_) were defined as nontoxic [39].

### Virus titre and genome detection

The virus solution to be detected was diluted serially 10-fold. Each concentration solution was added to each column at 100 μL per well, which was repeated 8 times for each concentration, followed by the addition of 100 μL of 2% MEM. The control was established in virus-free medium and cultured at 37 °C in 5% CO_2_. The virus TCID_50_ was calculated using the Reed and Muench formula. Relative RT-qPCR was used to evaluate FCV gene expression. Briefly, first, the RNA of the virus was extracted from the mixture of cells and medium (by freezing and thawing three times) with the Simply P Total RNA Extraction Kit (Bioflux, China); then, the RNA was reverse-transcribed into complementary DNA (cDNA) (Thermo, USA), followed by RT-qPCR using TB Green Premix Ex Taq II (Tli RNaseH Plus) (TaKaRa, China). The RT-qPCR conditions were based on the Applied Biosystems 7500 Fast Real-Time PCR System with TB Green Premix Ex Taq II (Tli RNaseH Plus) (TaKaRa, China). The upstream and downstream sequences of the FCV primers were 5′-GCAGGTTGGGATAAACATGGA-3′ and 5′-CACGAGGCGATTGAGTTGAG-3′, and for GAPDH, the sequences were 5′-TGGAAAGCCCATCACCATC-3′ and 5′-ACTCCACAACATACTCAGCACCA-3′.

### Antiviral effect of copper chloride on FCV

F81 cells at 100% confluence were infected with FCV CH-JL2 at 100 TCID_50_, while 200 μL of different concentrations of copper chloride (200–20 μM) were added to the medium. MEM containing 0.4% DMSO was used for the mock treatment group. After incubation for 28 h at 37 °C in 5% CO_2_, the TCID_50_ levels of the virus and the RT-qPCR results were determined to assess the antiviral effect on FCV. The half-maximal inhibitory concentration (IC_50_) of copper chloride for FCV was also determined, and the results were plotted using GraphPad Prism 8.

### Indirect immunofluorescence assay (IFA)

To more intuitively observe the antiviral effect of copper chloride on FCV, we performed an IFA [38]. As described above, a monolayer of F81 cells infected with 100 TCID_50_ of FCV was exposed to different concentrations of copper chloride and then fixed in 80% cold acetone for 30 min. Next, the cells were washed 5 times with PBS containing 0.05% Tween-20 (PBST). Then, primary antibody (VMRD, USA) diluted 300-fold with 1% BSA was added, and the cells were incubated for 1 h at 37 °C. The supernatant was discarded, the cells were washed 5 times with PBST, and 200-fold diluted FITC-labelled rabbit anti-cat secondary antibody (Bioss Antibody, China) was added to the cells in the dark and incubated at 37 °C for 1 h. The fluorescence was observed under an inverted Leica DMi8 fluorescence microscope (Leica, Germany), and the fluorescence results were analysed by greyscale scanning.

### Effect of different copper chloride treatment times on FCV

To determine whether the antiviral effect of copper chloride on F81 cells was time-dependent, copper chloride solution at a final concentration of 80 μM was administered − 1 h, 0 h, 2 h, 4 h, 6 h, 8 h, and 16 h after FCV infection. MEM containing 0.4% DMSO was used for the mock treatment group. After incubation for 24 h at 37 °C in 5% CO_2_, the TCID_50_ level was determined, and the RT-qPCR results were assessed to determine the amount of virus.

### Antiviral effect of copper chloride on different strains

To determine whether copper chloride also has antiviral effects on other FCV strains, we diluted different strains of FCV (CH-JL1, CH-JL3, CH-JL4, and CH-SH) to 100 TCID_50_ and infected F81 cells, which were subsequently treated with copper chloride solution at a final concentration of 80 μM. After incubating the cells with virus for 28 h at 37 °C in 5% CO_2_, the TCID_50_ level was determined, and the RT-qPCR results were assessed to determine the amount of virus.

### Combination of copper chloride and ribavirin or F (ab’)_2_

To evaluate the combined action of the compounds, we diluted the different drugs by the checkerboard method and mixed the two drugs thoroughly to determine the combined effect on FCV of different concentrations of copper chloride (0–40 μM) and ribavirin (0–15 μM)/F (ab’)_2_ (0–300 μg). The results from the RT-qPCR experiments were statistically analysed in a manner described previously, and the effects of the drug combinations were evaluated using SynergyFinder [31]. The zero-interaction efficiency (ZIP) model [25] was used to calculate the mutual scores of the different concentrations of the drug. For each treatment, at least three data sets from triplicate independent experiments were analysed. The results are expressed as the mean and standard error of the mean (SEM).

### Statistical methods

All experiments were performed three times independently in triplicate. The data are expressed as the mean ± standard deviation (SD). The significance of the differences between the groups were determined by paired t-tests and one-way/two-way analysis of variance.

## Data Availability

The datasets used and/or analysed during the current study are available from the corresponding author upon reasonable request.

## Abbreviations

PMO: Minomorpho oligophosphate
TCID_50_: 50% tissue culture infective dose
RT-qPCR: Real-time quantitative polymerase chain reaction
CC_50_: 50% cytostatic concentration
PBS: Phosphate-buffered saline
IC_50_: Half-maximal inhibitory concentration
IFA: Indirect immunofluorescence assay
SEM: Standard error of the mean
FITC: Fluorescein isothiocyanate
ZIP: Zero-interaction efficiency
F81 cells: Feline kidney fibroblast (F81) cells

## References

[1] Clarke IN, Lambden PR (1997). The molecular biology of caliciviruses. J Gen Virol.

[2] Di Martino B, Di Rocco C, Ceci C, Marsilio F (2009). Characterization of a strain of feline calicivirus isolated from a dog faecal sample. Vet Microbiol.

[3] Martella V, Pratelli A, Gentile M, Buonavoglia D, Decaro N, Fiorente P, Buonavoglia C (2002). Analysis of the capsid protein gene of a feline-like calicivirus isolated from a dog. Vet Microbiol.

[4] Battilani M, Vaccari F, Carelle MS, Morandi F, Benazzi C, Kipar A, Dondi F, Scagliarini A (2013). Virulent feline calicivirus disease in a shelter in Italy: a case description. Res Vet Sci.

[5] August JR (2006). Dedication. Consultations in Feline Internal Medicine (Fifth Edition).

[6] Sykes JE (2014). Pediatric feline upper respiratory disease. Vet Clin North Am Small Anim Pract.

[7] Hurley KF, Sykes JE (2003). Update on feline calicivirus: new trends. Vet Clin North Am Small Anim Pract.

[8] Cohn LA (2011). Feline respiratory disease complex. Vet Clin North Am Small Anim Pract.

[9] Willi B, Spiri AM, Meli ML, Samman A, Hoffmann K, Sydler T, Cattori V, Graf F, Diserens KA, Padrutt I (2016). Molecular characterization and virus neutralization patterns of severe, non-epizootic forms of feline calicivirus infections resembling virulent systemic disease in cats in Switzerland and in Liechtenstein. Vet Microbiol.

[10] Coyne KP, Gaskell RM, Dawson S, Porter CJ, Radford AD (2007). Evolutionary mechanisms of persistence and diversification of a calicivirus within endemically infected natural host populations. J Virol.

[11] Radford AD, Coyne KP, Dawson S, Porter CJ, Gaskell RM (2007). Feline calicivirus. Vet Res.

[12] Smith AW, Iversen PL, O'Hanley PD, Skilling DE, Christensen JR, Weaver SS, Longley K, Stone MA, Poet SE, Matson DO (2008). Virus-specific antiviral treatment for controlling severe and fatal outbreaks of feline calicivirus infection. Am J Vet Res.

[13] Wu H, Zhang X, Liu C, Liu D, Liu J, Tian J, Qu L (2015). Antiviral effect of lithium chloride on feline calicivirus in vitro. Arch Virol.

[14] Wu H, Liu Y, Zu S, Sun X, Liu C, Liu D, Zhang X, Tian J, Qu L (2016). In vitro antiviral effect of germacrone on feline calicivirus. Arch Virol.

[15] McDonagh P, Sheehy PA, Fawcett A, Norris JM (2015). Antiviral effect of mefloquine on feline calicivirus in vitro. Vet Microbiol.

[16] Debski B (2016). Supplementation of pigs diet with zinc and copper as alternative to conventional antimicrobials. Pol J Vet Sci.

[17] Horie M, Ogawa H, Yoshida Y, Yamada K, Hara A, Ozawa K, Matsuda S, Mizota C, Tani M, Yamamoto Y (2008). Inactivation and morphological changes of avian influenza virus by copper ions. Arch Virol.

[18] Shahabadi N, Abbasi AR, Moshtkob A, Shiri F (2019). DNA-binding studies of a new Cu (II) complex containing reverse transcriptase inhibitor and anti-HIV drug zalcitabine. J Coord Chem.

[19] Sucipto TH, Churrotin S, Setyawati H, Kotaki T, Martak F, Soegijanto S (2017). Antiviral activity of copper (ii) chloride DIHYDRATE against dengue virus TYPE-2 in VERO cell. Indones J Trop Infect Dis.

[20] Jopp M, Becker J, Becker S, Miska A, Gandin V, Marzano C, Schindler S (2017). Anticancer activity of a series of copper (II) complexes with tripodal ligands. Eur J Med Chem.

[21] Sagripanti JL, Routson LB, Bonifacino AC, Lytle CD (1997). Mechanism of copper-mediated inactivation of herpes simplex virus. Antimicrob Agents Chemother.

[22] Betanzos-Cabrera G, Rez FJR, Oz JLM, Barrn BL, Maldonado R (2004). Inactivation of HSV-2 by ascorbate-Cu (II) and its protecting evaluation in CF-1 mice against encephalitis. J Virol Methods.

[23] Wen-Jie G, Si-Si Y, Ning C, Juan H, Jing G, Qiu-Yun C. ROS-mediated autophagy was involved in cancer cell death induced by novel copper(II) complex. Experimental and toxicologic pathology: official journal of the Gesellschaft fur Toxikologische Pathologie. 2010;62(5):577–82. 10.1016/j.etp.2009.08.001 19758794

[24] Strauch BM, Niemand RK, Winkelbeiner NL, Hartwig A (2017). Comparison between micro- and nanosized copper oxide and water soluble copper chloride: interrelationship between intracellular copper concentrations, oxidative stress and DNA damage response in human lung cells. Part Fibre Toxicol.

[25] Yadav B, Wennerberg K, Aittokallio T, Tang J (2015). Searching for drug synergy in complex dose–response landscapes using an interaction potency model. Comput Struct Biotechnol J.

[26] Rifkind JM, Shin YA, Heim JM, Eichhorn GL (1976). Cooperative disordering of single-stranded polynucleotides through copper crosslinking. Biopolymers.

[27] Kopitz J, von Reitzenstein C, Muhl C, Cantz M (1994). Role of plasma membrane ganglioside sialidase of human neuroblastoma cells in growth control and differentiation. Biochem Biophys Res Commun.

[28] Miyagi T, Hata K, Hasegawa A, Aoyagi T (1993). Differential effect of various inhibitors on four types of rat sialidase. Glycoconj J.

[29] RAFELSON MJ, SCHNEIR M, WILSON VJ (1963). Studies on the neuraminidase of influenza virus. Ii. Additional properties of the enzymes from the Asian and PR 8 strains. Arch Biochem Biophys.

[30] Howe C, Morgan C (1969). Interactions between Sendai virus and human erythrocytes. J Virol.

[31] Ianevski A, He L, Aittokallio T, Tang J (2017). SynergyFinder: a web application for analyzing drug combination dose-response matrix data. Bioinformatics (Oxford, England).

[32] Glover ZK, Basa L, Moore B, Laurence JS, Sreedhara A (2015). Metal ion interactions with mAbs: Part 1.

[33] Smith MA, Easton M, Everett P, Lewis G, Payne M, Riveros-Moreno V, Allen G (1996). Specific cleavage of immunoglobulin G by copper ions. Int J Pept Protein Res.

[34] Belczyk-Ciesielska A, Zawisza IA, Mital M, Bonna A, Bal W (2014). Sequence-specific Cu (II)-dependent peptide bond hydrolysis: similarities and differences with the Ni (II)-dependent reaction. Inorg Chem.

[35] Protas AM, Bonna A, Kopera E, Bal W (2011). Selective peptide bond hydrolysis of cysteine peptides in the presence of Ni (II) ions. J Inorg Biochem.

[36] Salinas BA, Sathish HA, Shah AU, Carpenter JF, Randolph TW (2010). Buffer-dependent fragmentation of a humanized full-length monoclonal antibody. J Pharm Sci.

[37] Yanli Z, Xiaoqing C, Ying Y, Kai W, Hongwei D, Chao G, Songtao Y, Guixue H: Isolation and phylogenetic analysis of three feline calicivirus strains from domestic cats in Jilin Province, China. Arch Virol. 2017;162(9):2579–89. 10.1007/s00705-017-3392-3 28478577

[38] Cui Z, Li D, Yi S, Guo Y, Dong G, Niu J, Zhao H, Zhang Y, Zhang S, Cao L, et al. Equine immunoglobulin F (ab') 2 fragments protect cats against feline calicivirus infection. Int Immunopharmacol. 2019;75. 10.1016/j.intimp.2019.105714 PMC7106254 31352323

[39] Jasenosky LD, Cadena C, Mire CE, Borisevich V, Haridas V, Ranjbar S, Nambu A, Bavari S, Soloveva V, Sadukhan S (2019). The FDA-Approved Oral Drug Nitazoxanide Amplifies Host Antiviral Responses and Inhibits Ebola Virus. iScience.

